# Supplementary material for: Survival of polycyclic aromatic hydrocarbon knockout fragments in the interstellar medium
Source: Nat Commun. 2021 Nov 17;12:6646. doi: 10.1038/s41467-021-26899-0 (PMC8599666; doi:10.1038/s41467-021-26899-0)
Supplement: Supplementary file 2 — Description of Additional Supplementary Files [file 41467_2021_26899_MOESM2_ESM.docx]

**Description of Additional Supplementary Files**

**File Name:** Supplementary Movie 1

**Description:** Classical molecular dynamics simulation of a He atom colliding with a coronene molecule with a velocity of 72 km/s. See the methods section of the article for simulation details.
